# Demographic and geographical trends in chronic lower respiratory diseases mortality in the United States, 1999 to 2020

**DOI:** 10.1186/s12931-024-02880-5

**Published:** 2024-06-24

**Authors:** Nikita Baral, Ali Bin Abdul Jabbar, Amna Noor, Mohsin Mirza, Bradley DeVrieze, Alec Hildenbrand, Abubakar Tauseef

**Affiliations:** 1 Creighton University School of Medicine, Omaha, NE USA; 2 Department of Internal Medicine, Creighton University Medical Center, Omaha, NE USA; 3 Services Hospital, Lahore, Pakistan

**Keywords:** Chronic lower respiratory diseases, Mortality trends, CDC WONDER database, United States, Joinpoint regression, Disparities, Demographic, geographical

## Abstract

Chronic lower respiratory disease (CLRD) related mortality has decreased in the United States due to increasing awareness in the general population and advancing preventative efforts, diagnostic measures, and treatment. However, demographic and regional differences still persist throughout the United States. In this study, we analyzed the temporal trends of demographic and geographical differences in CLRD-related mortality. Data was extracted from the Centers for Disease Control and Prevention Wide-Ranging Online Data for Epidemiologic Research (CDC WONDER) database. Using this data, age-adjusted mortality rates per 100,000 people (AAMR), annual percentage change (APC), and average annual percentage changes with 95% confidence intervals (CIs) were assessed. The Joinpoint Regression Program was used to determine mortality trends between 1999 and 2020 based on demographic and regional groups.

During this study period, there were 3,064,049 CLRD-related deaths, with most demographics and regional areas showing an overall decreasing trend. However, higher mortality rates were seen in the non-Hispanic White population and rural areas. Interestingly, mortality rates witnessed a decreasing trend for males throughout the study duration compared to females, who only began to show decreases in mortality during the latter half of the 2010s. Using these results, one can target efforts and build policies to improve CLRD-related mortality and reduce disparities in the coming decades.

**Supplementary Information:**

The online version contains supplementary material available at 10.1186/s12931-024-02880-5.

## Introduction

In 2020, chronic lower respiratory diseases (CLRD) accounted for one of the five leading causes of mortality in the United States [1]. Based on the definitions by the Centers for Disease Control and Prevention (CDC) and the World Health Organization (WHO), CLRD encompasses a group of disorders that affect the lungs and airways, including chronic obstructive pulmonary disease (COPD), chronic bronchitis, emphysema, and asthma [2]. Risk factors associated with CLRD include tobacco use, air pollution, allergen exposures, and physical inactivity [3]. With these risk factors in mind, older individuals are more likely to be diagnosed with CLRD due to higher incidences of COPD and emphysema in these aging populations [4]. Although in other disease processes, minority ethnicities are disproportionately affected, COPD is one illness in which the non-Hispanic White population has higher rates of diagnosis [4].

Throughout the years, it is evident that the overall chronic lower respiratory disease-related mortality has significantly decreased due to advances in preventative measures, screening tools, and treatment of CLRD. The decline in cigarette smoking due to increased education regarding the detrimental health effects, as well as advances in screening and diagnostic (X-ray, CT, spirometry) measures and treatment (medications, supplemental oxygen, and pulmonary rehabilitation), have contributed to the decline in mortality [5, 6]. For instance, previously published literature has illustrated that patients that quit smoking experience substantial improvements in their pulmonary function tests in the first year after quitting, and these tests can predict a patient’s morbidity and mortality [4]. In addition, studies have reported that increased participation in pulmonary rehabilitation programs, even just one time, benefits patients with reduction of symptoms and improves quality of life [4]. Preventative vaccinations, such as influenza and pneumococcal, as well as the usage of antibiotics during exacerbations, have reduced subsequent death by around 50% in patients with CLRD [4].

Previous research has demonstrated an overall increase in CLRD AAMR nationally when comparing 1980 to 2014; however, recent reports show declines in AAMR towards the end of that study period [7]. While studies have shown significant variations in trends of CLRD-related mortality rates throughout the United States, an in-depth review regarding the differences in demographics (i.e., racial/ethnic groups, sex, and age groups), as well as regional areas with recent data from 2014 onward has not yet been studied [7]. Hence, to further understand these differences, this study utilized the Centers for Disease Control and Prevention Wide-Ranging Online Data for Epidemiologic Research (CDC WONDER) national database to analyze the death certificates of patients with CLRD-related mortality in the United States from 1999 to 2020.

## Methods

### Study design and database

Centers for Disease Control and Prevention Wide-ranging Online Data for Epidemiologic Research (CDC WONDER) was used to identify chronic lower respiratory-related deaths in the United States [8]. The Underlying Cause-of-Death Public Use Record and the CDC WONDER database death certificate records were analyzed to determine the chronic lower respiratory diseases-related cause of death as a contributing cause on nationwide death certificate records. The study was exempt from institutional review board approval because the CDC WONDER database contains anonymized, publicly available data.

We extracted data regarding chronic lower respiratory disease-related deaths and population sizes from 1999 to 2020. The International Classification of Diseases (ICD), 10th Revision, Clinical Modification codes used to analyze data regarding chronic lower respiratory disease-related mortality included J40-J47 (J41.0, J41.1, J41.8, J42, J43.0, J43.1, J43.2, J43.8, J43.9, J44.0, J44.1, J44.8, J44.9, J45.0, J45.1, J45.8, J45.9, J46, and J47) [9]. Common diagnoses included in these ICD codes include bronchitis, emphysema, chronic obstructive pulmonary disease, asthma, status asthmaticus, and bronchiectasis (Supplemental Table 1).

### Demographic and geographical study groups

Specifically, data extracted for analysis included gender, race/ethnicity, age groups, region, state, and urban-rural classification. Genders included males or females. Race/ethnicity groups were divided into non-Hispanic (NH) white, NH Black or African American, NH Asian or Pacific Islander, NH American Indian or Alaska Native, and Hispanic or Latino based on what was listed on the patient’s death certificate. Age groups included 25 to 39, 40 to 54, 55 to 69, 70 to 84, and 85 or older. For urban-rural classifications, the National Center for Health Statistics Urban-Rural Classification Scheme was used to divide the population into urban (large metropolitan area [population ≥ 1 million], medium/small metropolitan area [population 50,000 to 999,999]) and rural (population < 50,000) counties per the 2013 United States census classification [10]. Regions were classified into Northeast, Midwest, South, and West according to the Census Bureau definitions.

### Statistical analysis

Chronic lower respiratory disease-related crude mortality rate (CMR) and age-adjusted mortality rates (AAMR) were calculated. Crude mortality rates were calculated by dividing the number of CLRD-related deaths by the corresponding United States population. AAMR controls for the population’s variation in age distribution, allowing comparison of data, and was standardized using the 2000 United States standard population [11]. The Joinpoint Regression Program (Joinpoint version 4.9.0.0 available from National Cancer Institute, Bethesda, Maryland) was used to determine trends in mortality within the study period [12]. This program identifies significant changes in annual mortality trends over time through Joinpoint regression, which fits models of linear segments where significant temporal variation occurred. Annual percentage change (APC) with 95% confidence intervals (CIs) for the AAMRs were calculated for the line segments linking a Joinpoint using the Monte Carlo permutation test. The weighted average of the APCs was calculated and reported as AAPCs and corresponding 95% CIs to summarize the reported mortality trend for the entire study period. APC and AAPCs were considered increasing or decreasing if the slope describing the change in mortality over the time interval was significantly different from zero using a 2-tailed t-test. Statistical significance was set at *p* ≤ 0.05 (represented by asterisk ‘*’ in results and figures) [13].

## Results

### Overall

Between the years of 1999 and 2020, there were 3,064,049 reported deaths related to chronic lower respiratory diseases in this study population.

**Fig. 1:**
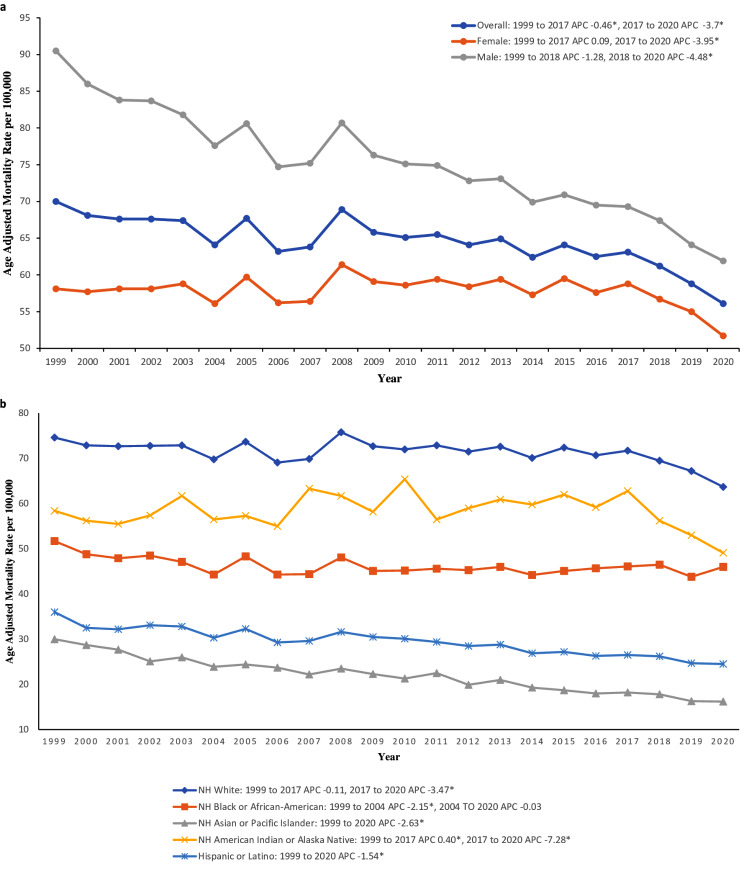
(**a**) Overall and gender stratified data regarding mortality rates associated with chronic lower respiratory diseases in the United States between 1999 and 2020. *Indicates the APC is significantly different from 0. (**b**) Stratified data by racial/ethnic groups regarding mortality rates associated with chronic lower respiratory diseases in the United States between 1999 and 2020. *Indicates the APC is significantly different from 0

Overall, the age-adjusted mortality rate (AAMR) decreased significantly from 70 (95% CI 69.6 to 70.4) in 1999 to 64.2 (95% CI 64.2 to 64.3) in 2020 with an AAPC of -0.93* (95% CI -1.08 to -0.76) (Supplemental Table 2; Supplemental Fig. 1). From 1999 to 2017, the APC in AAMR was − 0.46* (95% CI − 0.64 to -0.22), which then increased significantly from 2017 to 2020 to -3.70* (95% CI -6.04 to -1.86) (Fig. 1).

By the end of the study period, the highest mortality rate was seen in the elderly population (ages 85 or older), with a CMR of 612.6 in 2020. The largest mortality rates were also seen in non-Hispanic White males, with an AAMR OF 68.4 in 2020. Although males demonstrated the highest overall mortality in the study with declining trends, it is important to recognize that females, although having overall lower mortality, did not demonstrate the same declining trend until the latter half of the 2010s. Lastly, regionally, the males in both the Midwest and South had the greatest AAMR of 70.2 and 68.4, respectively. Males in rural populations had the highest mortality rate of all populations, with an AAMR of 87.4.

### Demographic differences

#### Gender stratified

In men, the AAMR decreased from 90.5 (95% CI 89.8 to 91.2) in 1999 to 61.9 (95% CI 61.4 to 62.3) in 2020, with an AAPC of -1.59* (95% CI -1.79 to -1.22) (Supplemental Table 2; Supplemental Fig. 1). The APC in AAMR was − 1.28 (95% CI -1.50 to 0.85) from 1999 to 2018, which then accelerated significantly from 2018 to 2020 to -4.48* (95% CI -6.65 to -1.45) (Fig. 1).

In women, the AAMR overall decreased from 58.1 (95% CI 57.6 to 58.5) in 1999 to 51.7 (95% CI 51.4 to 52.1) in 2020, with an AAPC of -0.49* (95% CI -0.74 to -0.25) (Supplemental Table 2; Supplemental Fig. 1). However, the APC in AAMR was 0.09 (95% CI -0.08 to 0.34) from 1999 to 2017, which finally trended downward from 2017 to 2020 to -3.95* (95% CI -7.52 to -1.51) (Fig. 1).

#### Race stratified

For chronic lower respiratory diseases, the highest mortality was seen in the non-Hispanic (NH) White population, which remained the highest throughout the years of 1999–2020. The AAMR decreased slightly from 74.6 (95% CI 74.1 to 75.0) in 1999 to 71.2 (95% CI 71.1 to 71.3) in 2020, with an AAPC of -0.60 (95% CI -0.84 to -0.34) (Supplemental Table 3; Supplemental Fig. 2a). The AAMR APC was − 0.11 (95% CI -0.29 to 0.17) from 1999 to 2017, which accelerated to -3.47* (95% CI -6.88 to -1.08) from 2017 to 2020 (Supplemental Fig. 2b). Non-Hispanic Asian or Pacific Islander populations remained as the lowest mortality racial group with an AAMR that decreased significantly from 30.0 (95% CI 28.1 to 31.8) in 1999 to 16.2 (95% CI 15.6 to 16.9) in 2020 with an APC of -2.63* (95% CI -2.90 to -2.33) (Supplemental Table 3; Supplemental Fig. 2a). Non-Hispanic Black or African American populations had a steady AAMR of 51.7 (95% CI 50.5 to 52.9) in 1999 to 46.0 (95% CI 45.1 to 46.8) in 2020. With an AAPC of -0.54 (95% CI -0.80 to -0.17), this population had a mortality rate between NH White, the highest AAMR racial group, and NH Asian or Pacific Islander, the lowest AAMR racial group (Supplemental Fig. 2b). The AAMR APC was − 2.15* (95% CI -6.15 to -0.48) from 1999 to 2004, which decelerated to -0.03 (95% CI -0.30 to 1.47) from 2004 to 2020 (Supplemental Fig. 2b).

#### Race and gender stratified

**Fig. 2:**
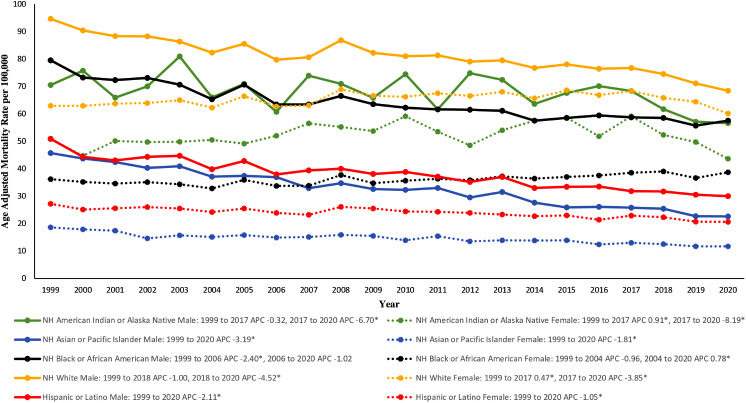
Data stratified by racial/ethnic groups and gender for chronic lower respiratory diseases related mortality in the United States between 1999 and 2020. *Indicates the APC is significantly different from 0

On further stratification of both gender and race, the highest AAMR was seen in NH White males with a profound decrease in AAMR of 94.6 (95% CI 93.8 to 95.4) in 1999 to 68.4 (95% CI 67.9 to 69.0) in 2020 (Fig. 2; Supplemental Table 4). With an AAPC of -1.33* (95% CI -1.57 to -0.97), the 1999–2018 APC was − 1.00 (95% CI -1.18 to 0.21), and the 2018–2020 APC accelerated to -4.52* (95% CI -7.04 to -1.17) (Supplemental Fig. 3a). NH Asian or Pacific Islander males had the lowest mortality rate with an APC of -3.19* (95% CI -3.45 to -2.88) (Supplemental Fig. 3a).

For females, NH White females had a significant increase in AAMR from 62.9 (95% CI 62.4 to 63.5) in 1999 to 68.3 (95% CI 67.8 to 68.8) in 2017, followed by a decrease in AAMR to 60.1 (95% CI 59.6 to 60.5) in 2020 (Fig. 2; Supplemental Table 4). The 1999–2017 APC was 0.47* (95% CI 0.26 to 0.74) before finally assuming a downward trend from 2017 to 2020 with an APC of -3.85* (95% CI -6.49 to -1.78) (Supplemental Fig. 3b). NH American Indian or Alaska Native females followed a similar trend with marked increase in AAMR from 50.8 (95% CI 43.6 to 58) in 1999 to 59.1 (95% CI 53.7 to 64.4) in 2017 (1999–2017 APC 0.91 (95% CI 0.43 to 1.70)), followed by a decreasing trend in AAMR to 43.6 (95% CI 39.3 to 47.8) by 2020 (2017–2020 APC − 8.19 (95% CI -16.28 to -3.30)) (Supplemental Fig. 3b). NH Black or African American females demonstrated a decrease in AAMR from 1999 to 2004 with an APC of -0.96 (95% CI -4.73 to 0.51); however, between 2004 and 2020, this population illustrated an increase in AAMR with an APC of 0.78* (95% CI 0.50 to 2.19) (Fig. 2, Supplemental Fig. 3b). NH Asian or Pacific Islanders females remained with the lowest mortality rate with an APC of -1.81* (95% CI -2.19 to -1.32) (Supplemental Fig. 3b).

#### Age group stratified

Patients who are 85 years of age or older had the highest crude mortality rate, with a slight increase from 646.0 (95% CI 638.2 to 653.7) in 1999 to 700.6 (95% CI 694.1 to 707) in 2017, before decreasing to 612.6 (95% CI 606.7 to 618.6) in 2020 (Supplemental Table 5; Supplemental Fig. 4a). The 1999–2017 APC was 0.40* (95% CI 0.18 to 0.72) and the 2017–2020 APC was − 4.05* (95% CI -7.65 to -1.51) (Supplemental Fig. 4b). Followed by the 85 years or older age group, patients between 70 and 84 years of age had the second highest crude mortality rate, which decreased from 326.2 (95% CI 323.8 to 328.6) to 230.2 (95% CI 228.6 to 231.9) between 1999 and 2020 (Supplemental Table 5; Supplemental Fig. 4a). With an AAPC of -1.53* (95% CI -1.78 to -1.28), the 1999–2016 APC was − 0.81* (95% CI -1.04 to -0.50) and the 2016–2020 APC was − 4.55* (95% CI -7.87 to -2.76) (Supplemental Fig. 4b).

### Regional variation

#### Census region-based differences

In 1999, AAMR was highest in the West at 75.7 (95% CI 74.8 to 76.6), followed by the Midwest at 71.4 (95% CI 70.6 to 72.2) and the South at 71.6 (95% CI 71 to 72.3), with the Northeast region having the lowest AAMR at 60.6 (95% CI 59.8 to 61.4) (Supplemental Table 6). The Midwest and South have remained stable between 1999 and 2017 with an APC of 0.08 (95% CI -0.16 to 0.63) in the Midwest and − 0.09 (95% CI -0.28 to 0.19) in the South (Supplemental Table 6; Supplemental Fig. 5a). However, within this same period, AAMR in the West has dropped significantly below the Midwest and South with an APC of -1.50 (95% CI -1.65 to -1.20) (Supplemental Fig. 5b). Between 2017 and 2020, there has been a regional decline in AAMR in all four regions. By the end of the study, the highest AAMR was seen in the Midwest at 63.0 (95% CI 62.4 to 63.7), followed closely by the South at 62.3 (95% CI 61.8 to 62.7), then the West at 49.4 (95% CI 48.8 to 50), with the Northeast region still having the lowest AAMR at 47.0 (95% CI 46.4 to 47.6) (Supplemental Fig. 5b).

#### Gender and census region-based differences

**Fig. 3:**
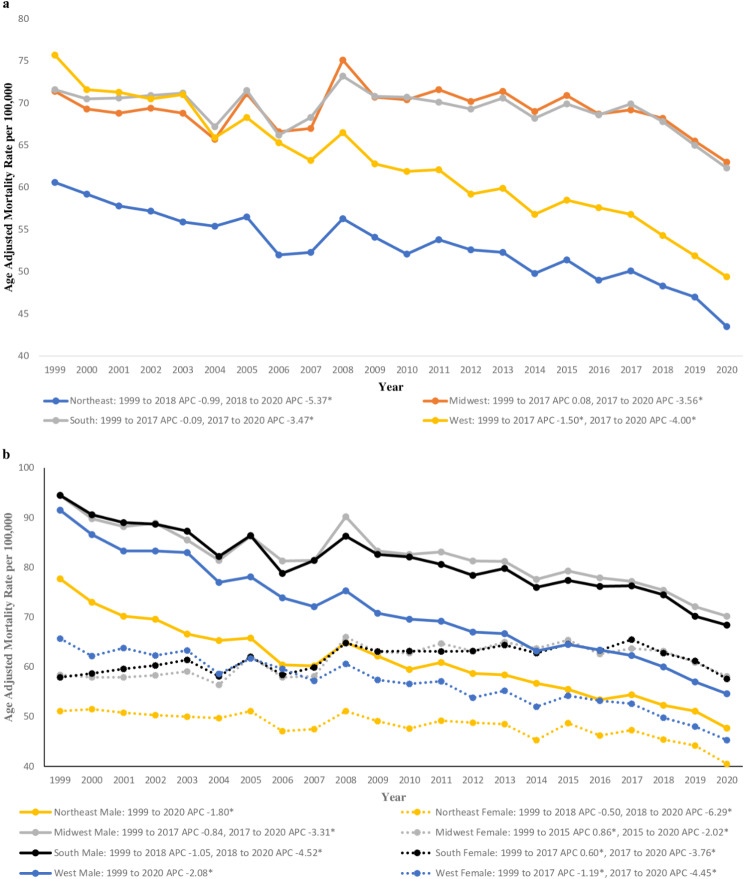
(**a**) Data stratified by region regarding mortality rates associated with chronic lower respiratory diseases in the United States between 1999 and 2020. *Indicates the APC is significantly different from 0. (**b**) Data stratified by region and gender regarding mortality rates associated with chronic lower respiratory diseases in the United States between 1999 and 2020. *Indicates the APC is significantly different from 0

Females in the Midwest region demonstrated an increase in AAMR from 58.4 (95% CI 57.5 to 59.4) in 1999 to 65.4 (95% CI 64.5 to 66.4) in 2015 (1999 to 2015 APC 0.86*), before decreasing to 58.1 (95% CI 57.2 to 58.9) in 2020 (2015 to 2020 APC − 2.02*) (Fig. 3; Supplemental Table 7; Supplemental Fig. 6a). Similar patterns were seen with females in the Southern region with increases in AAMR from 57.9 (95% CI 57.1 to 58.7) in 1999 to 64.6 (95% CI 63.9 to 65.3) in 2017 (1999 to 2017 APC 0.60*), before decreasing to 57.6 (95% CI 57.0 to 58.2) in 2020 (2017 to 2020 APC − 3.76*) (Fig. 3; Supplemental Table 7; Supplemental Fig. 6a). Although females in the West region began the study with the highest AAMR of 65.7 (95% CI 64.6 to 66.8), it decreased significantly to 45.3 (95% CI 44.6 to 46) by 2020 (1999 to 2017 APC − 1.19*, 2017 to 2020 APC − 4.45*) (Fig. 3; Supplemental Table 7; Supplemental Fig. 6a). Females in the Northeast region remained the lowest with an AAMR of 51.1 (95% CI 50.2 to 52) in 1999 to 40.5 (95% CI 39.7 to 41.2) in 2020 (Fig. 3; Supplemental Table 7; Supplemental Fig. 6a).

On average, in male populations throughout all of the regions, there were significant decreases in AAMR; however, in the Midwest and South, the male populations had higher AAMRs of 70.2 (95% CI 69.1 to 71.2) in the Midwest and 68.4 (95% CI 67.7 to 69.2) in the South (Fig. 3; Supplemental Table 7; Supplemental Fig. 6b). Northeast males had the lowest AAMR of 47.7 (95% CI 46.8 to 48.7) (Fig. 3; Supplemental Table 7; Supplemental Fig. 6b). The Midwest male population, although still decreasing in AAMR, had the lowest reduction in mortality with an AAPC of -1.20* (95% CI -1.45 to -0.83) compared to -1.38* (95% CI -1.66 to -0.90) in the Southern region, -1.80* (95% CI -2.01 to -1.59) in the Northeast region, and − 2.08* (95% CI -2.30 to -1.85) in the Western region (Fig. 3; Supplemental Table 7; Supplemental Fig. 6b).

#### State-level differences

Large state-to-state variations in AAMR exist within the United States. The lowest overall AAMR is 29.5 (95% CI 28.8 to 30.2) in Hawaii, while the highest AAMR is 95.1 (95% CI 94.1 to 96.1) in West Virginia (Supplemental Table 8). Other states included in the ≤ 10th percentile of chronic lower respiratory disease-related mortality include the District of Columbia (AAMR of 37.2), New Jersey (AAMR of 47.4), New York (AAMR of 47.9), Connecticut (AAMR of 50), and Utah (AAMR of 51.3). The states included in the ≥ 90th percentile of chronic lower respiratory disease-related mortality also include Indiana (AAMR of 83.7), Arkansas (AAMR of 85.5), Wyoming (AAMR of 89.7), Kentucky (AAMR of 94.1, Oklahoma (AAMR of 94.2), and West Virginia (AAMR of 95.1).

**Fig. 4:**
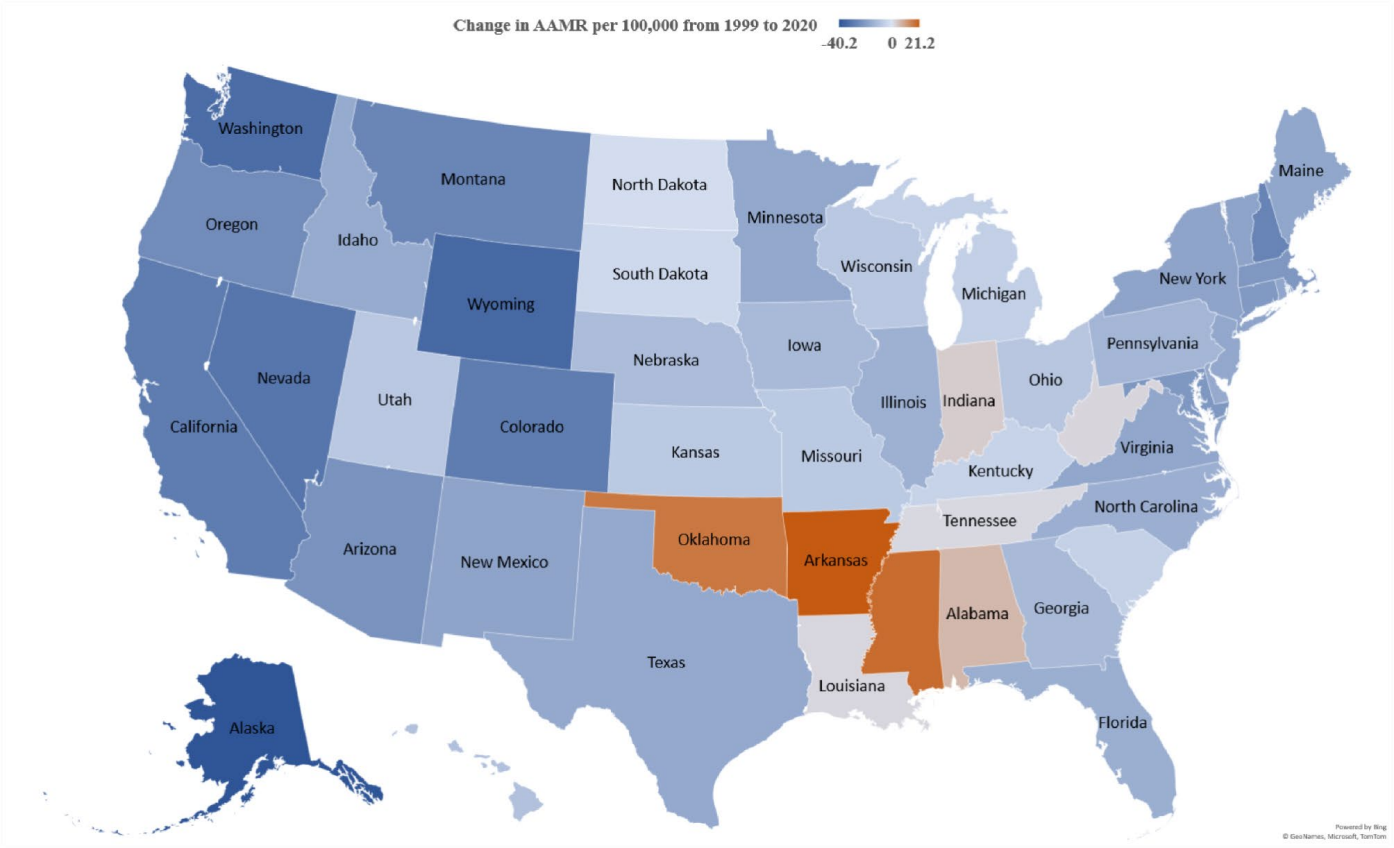
Data stratified by state regarding total change in AAMR per 100,000 from 1999 to 2020 for chronic lower respiratory diseases-related mortality in the United States

The state of Alaska had the largest decreased rate of change when comparing the AAMR from 1999 to 2020 of -40.2 with an APC of -2.02* (95% CI -2.56 to -1.43) (Supplemental Table 9; Supplemental Fig. 7a, 8a). Followed by Wyoming and Washington, with a decrease in AAMR of -34.5 (AAPC of -1.06* (95% CI -1.59 to -0.50)) in Wyoming, and − 33.3 (AAPC of -2.36* (95% CI -2.91 to -1.91)) in Washington (Supplemental Table 10; Supplemental Fig. 7b). Arkansas had the most increase in AAMR by 21.2 (AAPC of 1.29* (95% CI 0.89 to 1.90)) (Supplemental Table 10; Supplemental Fig. 8a, 8b). Hawaii demonstrated the lowest AAMR throughout 1999–2020 decreasing from 36.9 to 27.4 with a change of AAMR of -9.5 from 1999 to 2020 (Fig. 4).

#### Rural versus urban differences

**Fig. 5:**
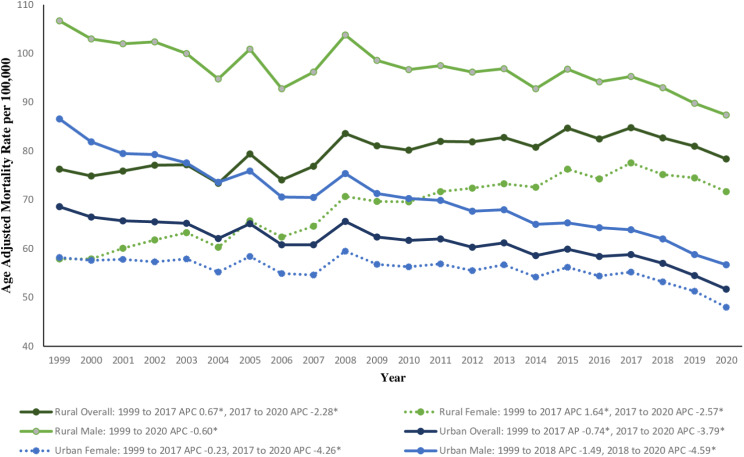
Data stratified by gender and urban-rural classifications for chronic lower respiratory diseases related mortality in the United States between 1999 and 2020. *Indicates the APC is significantly different from 0

Throughout the study period, urban populations had average AAMRs that were lower at 51.7 (95% CI 51.4 to 52) compared to rural areas at 78.4 (95% CI 77.6 to 79.2) (Fig. 5, Supplemental Table 11). Overall, the urban population decreased in AAMR with an AAPC of -1.18* (95% CI -1.44 to -0.90) compared to rural areas at an AAPC of 0.25* (95% CI 0.03 to 0.49). In 1999, urban females and rural females had similar AAMRs, with urban females at 58.2 (95% CI 57.7 to 58.7) and rural females at 57.9 (95% CI 56.8 to 58.9). However, the difference widened with urban females overall having a decrease in mortality with an AAPC of -0.81* (95% CI -1.06 to -0.52), the 1999–2017 APC was − 0.23 (95% CI -0.43 to -0.08) and the 2017–2020 APC was − 4.26* (95% CI -7.74 to -1.80) (Supplemental Fig. 9). In contrast, rural females had a slight increase in mortality with an AAPC of 1.03* (95% CI 0.76 to 1.27), the 1999–2017 APC was 1.64* (95% CI 1.44 to 1.92) and the 2017–2020 APC was − 2.57* (95% CI -6.23 to -0.23) (Supplemental Fig. 9). Both urban and rural males demonstrated decreases in mortality throughout the study period with AAPCs of -1.79* (95% CI -2.00 to -1.44) in urban males and − 0.60* (95% CI -0.79 to -0.38) in rural males (Supplemental Fig. 9).

## Discussion

In this study, there were several important findings regarding chronic lower respiratory diseases related mortality in the United States between the years of 1999–2020. These findings include: the overall mortality rates continuously decreased in last two decades; however, national mortality in females did not assume a downward trend until 2017. Additionally, the racial/ethnic group that demonstrated the highest mortality in the study period was the non-Hispanic White population. Lastly, state and regional variations in AAMR were seen, as well as, differences in mortality when comparing urban and rural counties.

Several factors have been suspected to contribute to the overall decrease in CLRD-related mortality from 1999 to 2020. Agreeing with previously published literature, increased tobacco control with the regulation of cigarette smoking advertisements and labelling of tobacco products may have contributed to the national decline of patient populations that smoke [14, 15]. With more education on the dangers of air pollution, the nation has seen advances in enhancing air quality [15–17]. Higher-risk occupational groups such as mining, construction, and healthcare have improved safety measures to exposures of toxins or hazards [18, 19]. There has also been an increase in preventative efforts to reduce the risk factors associated with CLRD. During annual appointments with primary care physicians, patients are educated on the importance of tobacco cessation and avoidance of certain triggers that may aggravate CLRD exacerbations [20]. Clinical pathways can also aid physicians in screening for those at risk of CLRD, allowing early diagnosis with spirometry and imaging and early treatment with medications such as corticosteroids or beta agonists [12, 13].

Although overall CLRD-related mortality has been decreasing in the United States, female mortality did not start to decrease until 2017. This is consistent with the previously published data that illustrates cigarette smoking first quickly declined among males starting in the 1960s. However, females did not demonstrate the same pattern until the 1980s [21]. Hence, due to this delay, females continued to smoke for longer periods compared to males, leading to increased CLRD as well as lung cancer diagnoses [22, 23]. Women, compared to their male counterparts, also tend to face challenges in the healthcare system with higher rates of misdiagnosis or delayed diagnosis, leading to inadequate and improper treatment [21].

Interestingly, in many of the other leading causes of mortality in the United States, African American populations lead with the highest mortality in the nation [24]. However, in this study, the highest mortality throughout the study was the NH White population. The results of this study demonstrating highest mortality in the NH White population, and not in the African American population, is conflicting with previous published findings. It may be due to competing risks, such as increased mortality by another reason unrelated to CLRD in African American populations. For instance, African American populations have the highest cardiovascular diseases-related mortality rate in the nation [25].

When analyzing racial groups with gender, all racial/ethnic groups in the male category decreased in mortality from 1999 to 2020. However, in female groups, NH White females and NH American Indian or Alaska Native females increased in AAMR until 2017, when they began to decrease. NH Black or African American women showed decreased mortality until 2004 when the mortality rate began to rise again. These racial differences may correlate to the race-based correction seen in diagnostic measures, such as spirometry. With this, the correction assumes that compared to a White patient, Black or African American patients have 10–15% smaller lung capacities, and Asian patients have 4–6% smaller lung capacities [26]. By assuming differences in lung capacity solely on racial background, patients of non-White backgrounds may receive false-negative results, missed diagnoses, and be deprived of the typical treatment of CLRD [27]. Furthermore, since over 10% of the population identifies with more than one racial or ethnic background, this correction does not allow for the adjustment to account for the diverse population of patients [26, 27].

Regional and geographic variations in CLRD-related mortality persist throughout the United States, agreeing with previously published literature. In 1999, the Western region led the nation in CLRD-related mortality; however, as the study progressed, this region trended below the Midwest and Southern regions. The Southern and Midwest regions still have elevated AAMRs compared to the other regional counterparts, which can potentially be explained by the presence of more risk factors such as air pollution, allergen exposures, and physical inactivity, as well as a high prevalence of tobacco use [28]. Like the results of gender differences in overall CLRD-related mortality, these regions demonstrate that females kept the AAMR trend upward. At the same time, male mortality decreased throughout all regions during the study period. State-by-state trends illustrate that throughout the 21–year study period, Alaska had the highest change in AAMR, while Hawaii had the lowest overall mortality. It is important to note that Arkansas saw increased mortality within the study period [28]. These results could be due to geographical locations, racial/ethnic prevalence, economic challenges, and risk factors prevalent in each of the states.

The results of this study demonstrated that overall, both rural and urban areas had downward trends of CLRD-related mortality. Urban areas had persistent decreases throughout the study period in both females and males. However, rural areas observed a rise in AAMR until 2017, then started to decrease. During this period, rural males continuously demonstrated decreased mortality throughout the study period. Yet, the rural female category AAMR increased, which increased overall rural mortality. This finding is consistent with previous studies demonstrating increased mortality in rural regions due to reduced access to care (cost, hospitals, specialty clinics, and physicians) and greater exposure to agricultural toxins and indoor air pollution [14, 28–31]. It is important to note that in both categories of rural and urban populations, patients with chronic lower respiratory diseases were not associated with a greater mortality rate from the COVID-19 pandemic in 2020, as the majority of these deaths occurred due to sepsis and septic shock related to the infection itself [32, 33].

Due to CDC WONDER data being collected from a public health database, this study may have some limitations. Variables such as social determinants of health could contribute to the patient’s death and were not reported on the website or death certificate. Furthermore, we only used the mortality data for chronic lower respiratory diseases as the underlying cause of death, and this does not take into account the deaths where chronic lower respiratory diseases might have contributed indirectly to mortality or acted as a secondary cause.

In conclusion, overall mortality related to chronic lower respiratory diseases has decreased significantly from 1999 to 2020. However, the results of this study demonstrated continued differences in mortality associated with gender, racial/ethnic backgrounds, and regional areas. To increase health equity throughout the United States, it is imperative to consider the differences and contemporary trends for future policymaking and resource allocation to minimize these differences in mortality trends and improve healthcare outcomes among all population groups.

### Electronic supplementary material

Below is the link to the electronic supplementary material.

Supplementary Material 1

## Data Availability

Data from the Centers for Disease Control and Prevention Wide-Ranging Online Data for Epidemiologic Research (CDC WONDER) website was used for this study. https://wonder.cdc.gov/. Data is provided within the manuscript and within the supplementary files.
