# Supplementary material for: Demographic and geographical trends in chronic lower respiratory diseases mortality in the United States, 1999 to 2020
Source: Respir Res. 2024 Jun 24;25:258. doi: 10.1186/s12931-024-02880-5 (PMC11197268; doi:10.1186/s12931-024-02880-5)

**Supplemental Figures and Tables:**

**Supplemental Table 1.** A list of ICD 10 codes used in this study.

| **ICD 10 codes** |
| --- |
| J40 (Bronchitis, not specified as acute or chronic) |
| J41 (Simple and mucopurulent chronic bronchitis) |
| J41.0 (Simple chronic bronchitis) |
| J41.1 (Mucopurulent chronic bronchitis) |
| J41.8 (Mixed uncomplicated and mucopurulent chronic bronchitis) |
| J42 (Unspecified chronic bronchitis) |
| J43 (Emphysema) |
| J43.0 (MacLeod syndrome) |
| J43.1 (Panlobular emphysema) |
| J43.2 (Centrilobular emphysema) |
| J43.8 (Other emphysema) |
| J43.9 (Emphysema, unspecified) |
| J44 (Other chronic obstructive pulmonary disease) |
| J44.0 (Chronic obstructive pulmonary disease with acute lower respiratory infection) |
| J44.1 (Chronic obstructive pulmonary disease with acute exacerbation, unspecified) |
| J44.8 (Other specified chronic obstructive pulmonary disease) |
| J44.9 (Chronic obstructive pulmonary disease, unspecified) |
| J45 (Asthma) |
| J45.0 (Predominantly allergic asthma) |
| J45.1 (Nonallergic asthma) |
| J45.8 (Mixed asthma) |
| J45.9 (Asthma, unspecified) |
| J46 (Status asthmaticus) |
| J47 (Bronchiectasis) |

**Supplemental Table 2.** Chronic lower respiratory diseases related mortality rates stratified by gender in the United States.

|  | **Age Adjusted Rate** | | |
| --- | --- | --- | --- |
| **Year** | **Overall** | **Female** | **Male** |
| 1999 | 70 | 58.1 | 90.5 |
| 2000 | 68.1 | 57.7 | 86 |
| 2001 | 67.6 | 58.1 | 83.8 |
| 2002 | 67.6 | 58.1 | 83.7 |
| 2003 | 67.4 | 58.8 | 81.8 |
| 2004 | 64.1 | 56.1 | 77.6 |
| 2005 | 67.7 | 59.7 | 80.6 |
| 2006 | 63.2 | 56.2 | 74.7 |
| 2007 | 63.8 | 56.4 | 75.2 |
| 2008 | 68.9 | 61.4 | 80.7 |
| 2009 | 65.8 | 59.1 | 76.3 |
| 2010 | 65.1 | 58.6 | 75.1 |
| 2011 | 65.5 | 59.4 | 74.9 |
| 2012 | 64.1 | 58.4 | 72.8 |
| 2013 | 64.9 | 59.4 | 73.1 |
| 2014 | 62.4 | 57.3 | 69.9 |
| 2015 | 64.1 | 59.5 | 70.9 |
| 2016 | 62.5 | 57.6 | 69.5 |
| 2017 | 63.1 | 58.8 | 69.3 |
| 2018 | 61.2 | 56.7 | 67.4 |
| 2019 | 58.8 | 55 | 64.1 |
| 2020 | 56.1 | 51.7 | 61.9 |
| **Number of Joinpoints (years of Joinpoint)** | 1 (2017) | 1 (2017) | 1 (2018) |
| **APC-Segment 1 (95% CI)** | -0.46 | 0.09 | -1.28 |
| **APC-Segment 2 (95% CI)** | -3.7 | -3.95 | -4.48 |
| **Average APC** | -0.93 | -0.49 | -1.59 |

**Supplemental Table 3.** Chronic lower respiratory diseases related mortality rates stratified by race in the United States.

| **Year** | **NH White** | **NH Black or African-American** | **NH Asian or Pacific Islander** | **NH American Indian or Alaska Native** | **Hispanic or Latino** |
| --- | --- | --- | --- | --- | --- |
| 1999 | 74.6 | 51.7 | 30 | 58.4 | 36 |
| 2000 | 72.9 | 48.8 | 28.7 | 56.2 | 32.5 |
| 2001 | 72.7 | 47.9 | 27.7 | 55.5 | 32.2 |
| 2002 | 72.8 | 48.5 | 25.1 | 57.4 | 33.1 |
| 2003 | 72.9 | 47.1 | 26 | 61.7 | 32.8 |
| 2004 | 69.8 | 44.3 | 23.9 | 56.5 | 30.3 |
| 2005 | 73.7 | 48.3 | 24.4 | 57.3 | 32.3 |
| 2006 | 69.1 | 44.3 | 23.7 | 55 | 29.3 |
| 2007 | 69.9 | 44.4 | 22.2 | 63.3 | 29.6 |
| 2008 | 75.8 | 48.1 | 23.5 | 61.7 | 31.6 |
| 2009 | 72.7 | 45.1 | 22.3 | 58.2 | 30.5 |
| 2010 | 72 | 45.2 | 21.3 | 65.4 | 30.1 |
| 2011 | 72.9 | 45.6 | 22.5 | 56.5 | 29.4 |
| 2012 | 71.5 | 45.3 | 19.9 | 59 | 28.5 |
| 2013 | 72.6 | 46 | 21 | 60.9 | 28.8 |
| 2014 | 70.1 | 44.2 | 19.3 | 59.8 | 26.9 |
| 2015 | 72.4 | 45.1 | 18.7 | 62 | 27.2 |
| 2016 | 70.7 | 45.7 | 18 | 59.2 | 26.3 |
| 2017 | 71.7 | 46.1 | 18.2 | 62.8 | 26.5 |
| 2018 | 69.5 | 46.5 | 17.8 | 56.2 | 26.2 |
| 2019 | 67.2 | 43.8 | 16.3 | 53 | 24.7 |
| 2020 | 63.7 | 46 | 16.2 | 49.1 | 24.5 |
| **Number of Joinpoints (years of Joinpoint)** | 1 (2017) | 1 (2004) | 0 | 1 (2017) | 0 |
| **APC-Segment 1 (95% CI)** | -0.11 | -2.15 | -2.63 | 0.4 | -1.54 |
| **APC-Segment 2 (95% CI)** | -3.47 | -0.03 |  | -7.28 |  |
| **Average APC** | -0.6 | -0.54 | -2.63 | -0.74 | -1.54 |

**Supplemental Table 4.** Chronic lower respiratory diseases related mortality rates stratified by race and gender in the United States.

| Year | NH American Indian or Alaska Native Male | NH American Indian or Alaska Native Female | NH Asian or Pacific Islander Male | NH Asian or Pacific Islander Female | NH Black or African American Male | NH Black or African American Female | NH White Male | NH White Female | Hispanic or Latino Male | Hispanic or Latino Female |
| --- | --- | --- | --- | --- | --- | --- | --- | --- | --- | --- |
| 1999 | 70.5 | 50.8 | 45.7 | 18.6 | 79.5 | 36.2 | 94.6 | 62.9 | 50.9 | 27.2 |
| 2000 | 75.7 | 44.7 | 43.7 | 17.9 | 73.2 | 35.2 | 90.4 | 62.9 | 44.3 | 25.1 |
| 2001 | 65.9 | 50.1 | 42.4 | 17.4 | 72.3 | 34.6 | 88.3 | 63.7 | 43 | 25.6 |
| 2002 | 70 | 49.7 | 40.3 | 14.6 | 73.1 | 35.1 | 88.2 | 63.9 | 44.3 | 26 |
| 2003 | 80.9 | 49.8 | 40.9 | 15.7 | 70.6 | 34.3 | 86.3 | 65 | 44.7 | 25.5 |
| 2004 | 66 | 50.5 | 37.1 | 15.1 | 65.3 | 32.8 | 82.3 | 62.2 | 39.8 | 24.2 |
| 2005 | 70.9 | 49.1 | 37.4 | 15.8 | 70.6 | 35.9 | 85.5 | 66.4 | 42.8 | 25.5 |
| 2006 | 60.7 | 52 | 36.9 | 14.9 | 63.4 | 33.7 | 79.7 | 62.7 | 37.9 | 23.9 |
| 2007 | 73.9 | 56.5 | 32.9 | 15.1 | 63.4 | 33.8 | 80.6 | 63.1 | 39.4 | 23.2 |
| 2008 | 70.9 | 55.2 | 34.7 | 15.9 | 66.5 | 37.7 | 86.8 | 68.9 | 40 | 26.1 |
| 2009 | 65.8 | 53.7 | 32.6 | 15.5 | 63.5 | 34.7 | 82.2 | 66.6 | 38.1 | 25.5 |
| 2010 | 74.4 | 59.1 | 32.3 | 13.9 | 62.2 | 35.6 | 81 | 66.2 | 38.8 | 24.4 |
| 2011 | 61.6 | 53.5 | 33 | 15.4 | 61.6 | 36.3 | 81.3 | 67.5 | 37.1 | 24.3 |
| 2012 | 74.8 | 48.5 | 29.5 | 13.5 | 61.5 | 35.8 | 79 | 66.5 | 35.2 | 23.9 |
| 2013 | 72.4 | 54 | 31.5 | 13.9 | 61.1 | 37.2 | 79.5 | 68 | 37 | 23.3 |
| 2014 | 63.6 | 57.5 | 27.6 | 13.8 | 57.5 | 36.4 | 76.7 | 65.7 | 33 | 22.7 |
| 2015 | 67.6 | 58.6 | 25.9 | 13.9 | 58.5 | 37 | 78 | 68.6 | 33.4 | 23 |
| 2016 | 70.1 | 51.8 | 26.1 | 12.4 | 59.4 | 37.5 | 76.4 | 66.8 | 33.5 | 21.4 |
| 2017 | 68.3 | 59.1 | 25.8 | 13 | 58.7 | 38.5 | 76.7 | 68.3 | 31.8 | 22.9 |
| 2018 | 61.7 | 52.3 | 25.4 | 12.5 | 58.5 | 39 | 74.5 | 65.8 | 31.7 | 22.3 |
| 2019 | 57.1 | 49.7 | 22.7 | 11.7 | 55.7 | 36.6 | 71.1 | 64.4 | 30.5 | 20.7 |
| 2020 | 56.6 | 43.6 | 22.6 | 11.7 | 57.5 | 38.7 | 68.4 | 60.1 | 30 | 20.6 |
| **Number of Joinpoints (years of Joinpoint)** | 1 (2017) | 1 (2017) | 0 | 0 | 1 (2006) | 1 (2004) | 1 (2018) | 1 (2017) | 0 | 0 |
| **APC-Segment 1 (95% CI)** | -0.32 | 0.91 | -3.19 | -1.81 | -2.4 | -0.96 | -1 | 0.47 | -2.11 | -1.05 |
| **APC-Segment 2 (95% CI)** | -6.7 | -8.19 |  |  | -1.02 | 0.78 | -4.52 | -3.85 |  |  |
| **Average APC** | -1.26 | -0.44 | -3.19 | -1.81 | -1.48 | 0.36 | -1.57 | -0.16 | -2.11 | -1.05 |

**Supplemental Table 5.** Chronic lower respiratory diseases related mortality rates stratified by age groups in the United States.

|  | **Crude Mortality Rate** | | | | |
| --- | --- | --- | --- | --- | --- |
| **Year** | **Age 25-39** | **Age 40-54** | **Age 55-69** | **Age 70-84** | **Age ≥85** |
| 1999 | 1 | 6.3 | 71.2 | 326.2 | 646 |
| 2000 | 1 | 6.4 | 66.8 | 316 | 648.6 |
| 2001 | 1 | 6.3 | 66.4 | 311.3 | 658.3 |
| 2002 | 1 | 6.6 | 62.4 | 314.3 | 670.3 |
| 2003 | 1 | 6.6 | 63.1 | 312.5 | 670.2 |
| 2004 | 0.9 | 6.4 | 58.6 | 299.5 | 643.2 |
| 2005 | 0.9 | 7.1 | 60.5 | 314.7 | 691.9 |
| 2006 | 0.8 | 6.9 | 56.4 | 294.5 | 641.3 |
| 2007 | 0.8 | 7.2 | 56.1 | 296.7 | 652 |
| 2008 | 0.8 | 7.5 | 60.3 | 318.9 | 722.7 |
| 2009 | 0.9 | 7.8 | 58.3 | 301.5 | 684.9 |
| 2010 | 0.9 | 7.4 | 57.1 | 297.3 | 690.7 |
| 2011 | 0.8 | 7.8 | 57.1 | 297.6 | 697.9 |
| 2012 | 0.8 | 7.7 | 56.8 | 288.9 | 687.8 |
| 2013 | 0.9 | 7.9 | 57.6 | 290.4 | 699.3 |
| 2014 | 1 | 7.7 | 57 | 275.9 | 670.5 |
| 2015 | 0.8 | 7.6 | 58.8 | 281.3 | 705.1 |
| 2016 | 0.9 | 7.6 | 58.9 | 273.8 | 676.9 |
| 2017 | 0.9 | 7.2 | 59.8 | 269.6 | 700.6 |
| 2018 | 0.8 | 6.9 | 59.4 | 258.5 | 682.4 |
| 2019 | 0.9 | 6.6 | 58.9 | 244.9 | 654.3 |
| 2020 | 1 | 6.7 | 58.2 | 230.2 | 612.6 |
| **Number of Joinpoints (years of Joinpoint)** | Not analyzed | 1 (2013) | 1 (2006) | 1 (2016) | 1 (2017) |
| **APC-Segment 1 (95% CI)** |  | 1.83 | -2.84 | -0.81 | 0.4 |
| **APC-Segment 2 (95% CI)** |  | -2.7 | 0.26 | -4.55 | -4.05 |
| **Average APC** |  | 0.3 | -0.79 | -1.53 | -0.24 |

**Supplemental Table 6.** Chronic lower respiratory diseases related mortality rates stratified by region in the United States.

| Year | Northeast | Midwest | South | West |
| --- | --- | --- | --- | --- |
| 1999 | 60.6 | 71.4 | 71.6 | 75.7 |
| 2000 | 59.2 | 69.3 | 70.5 | 71.6 |
| 2001 | 57.8 | 68.8 | 70.6 | 71.3 |
| 2002 | 57.2 | 69.4 | 70.9 | 70.5 |
| 2003 | 55.9 | 68.8 | 71.2 | 71 |
| 2004 | 55.4 | 65.7 | 67.2 | 65.9 |
| 2005 | 56.5 | 71.1 | 71.5 | 68.3 |
| 2006 | 52 | 66.6 | 66.2 | 65.3 |
| 2007 | 52.3 | 67 | 68.3 | 63.2 |
| 2008 | 56.3 | 75.1 | 73.2 | 66.5 |
| 2009 | 54.1 | 70.7 | 70.8 | 62.8 |
| 2010 | 52.1 | 70.4 | 70.7 | 61.9 |
| 2011 | 53.8 | 71.6 | 70.1 | 62.1 |
| 2012 | 52.6 | 70.2 | 69.3 | 59.2 |
| 2013 | 52.3 | 71.4 | 70.6 | 59.9 |
| 2014 | 49.8 | 69 | 68.2 | 56.8 |
| 2015 | 51.4 | 70.9 | 69.9 | 58.5 |
| 2016 | 49 | 68.7 | 68.6 | 57.6 |
| 2017 | 50.1 | 69.2 | 69.9 | 56.8 |
| 2018 | 48.3 | 68.2 | 67.8 | 54.3 |
| 2019 | 47 | 65.5 | 65 | 51.9 |
| 2020 | 43.5 | 63 | 62.3 | 49.4 |
| **Number of Joinpoints (years of Joinpoint)** | 1 (2018) | 1 (2017) | 1 (2017) | 1 (2017) |
| **APC-Segment 1 (95% CI)** | -0.99 | 0.08 | -0.09 | -1.5 |
| **APC-Segment 2 (95% CI)** | -5.37 | -3.56 | -3.47 | -4 |
| **Average APC** | -1.41 | -0.45 | -0.58 | -1.9 |

**Supplemental Table 7.** Chronic lower respiratory diseases related mortality rates stratified by region and gender in the United States.

| Year | Northeast Male | Northeast Female | Midwest Male | Midwest Female | South Male | South Female | West Male | West Female |
| --- | --- | --- | --- | --- | --- | --- | --- | --- |
| 1999 | 77.7 | 51.1 | 94.5 | 58.4 | 94.5 | 57.9 | 91.5 | 65.7 |
| 2000 | 73 | 51.5 | 89.8 | 57.9 | 90.6 | 58.7 | 86.6 | 62.2 |
| 2001 | 70.2 | 50.8 | 88.2 | 57.9 | 89 | 59.6 | 83.3 | 63.8 |
| 2002 | 69.6 | 50.3 | 88.9 | 58.3 | 88.7 | 60.3 | 83.3 | 62.3 |
| 2003 | 66.6 | 50 | 85.5 | 59.1 | 87.3 | 61.4 | 83 | 63.3 |
| 2004 | 65.3 | 49.7 | 81.4 | 56.4 | 82.2 | 58.1 | 77 | 58.6 |
| 2005 | 65.8 | 51.1 | 86.3 | 62.1 | 86.4 | 62 | 78.1 | 61.7 |
| 2006 | 60.4 | 47.1 | 81.3 | 57.9 | 78.8 | 58.4 | 73.9 | 59.6 |
| 2007 | 60.2 | 47.5 | 81.4 | 58.1 | 81.4 | 59.9 | 72.1 | 57.2 |
| 2008 | 64.9 | 51.1 | 90.2 | 66 | 86.3 | 64.8 | 75.3 | 60.6 |
| 2009 | 62.2 | 49.1 | 83.3 | 63 | 82.6 | 63.1 | 70.8 | 57.4 |
| 2010 | 59.5 | 47.6 | 82.6 | 62.8 | 82.1 | 63.2 | 69.6 | 56.6 |
| 2011 | 60.9 | 49.2 | 83.1 | 64.7 | 80.6 | 63.1 | 69.2 | 57.1 |
| 2012 | 58.7 | 48.8 | 81.3 | 63.2 | 78.4 | 63.2 | 67 | 53.8 |
| 2013 | 58.4 | 48.5 | 81.2 | 65 | 79.8 | 64.4 | 66.7 | 55.2 |
| 2014 | 56.7 | 45.3 | 77.6 | 63.7 | 76 | 62.8 | 63.2 | 52 |
| 2015 | 55.5 | 48.7 | 79.3 | 65.4 | 77.4 | 64.6 | 64.5 | 54.2 |
| 2016 | 53.4 | 46.2 | 77.9 | 62.6 | 76.2 | 63.3 | 63.4 | 53.2 |
| 2017 | 54.4 | 47.3 | 77.2 | 63.7 | 76.3 | 65.5 | 62.3 | 52.6 |
| 2018 | 52.3 | 45.4 | 75.4 | 63.2 | 74.5 | 62.8 | 60 | 49.8 |
| 2019 | 51.1 | 44.2 | 72.1 | 60.9 | 70.2 | 61.2 | 57 | 48 |
| 2020 | 47.7 | 40.5 | 70.2 | 58.1 | 68.4 | 57.6 | 54.6 | 45.3 |
| **Number of Joinpoints (years of Joinpoint)** | 0 | 1 (2018) | 1 (2017) | 1 (2015) | 1 (2018) | 1 (2017) | 0 | 1 (2017) |
| **APC-Segment 1 (95% CI)** | -1.8 | -0.5 | -0.84 | 0.86 | -1.05 | 0.6 | -2.08 | -1.19 |
| **APC-Segment 2 (95% CI)** |  | -6.29 | -3.31 | -2.02 | -4.52 | -3.76 |  | -4.45 |
| **Average APC** | -1.8 | -1.06 | -1.2 | 0.17 | -1.38 | -0.04 | -2.08 | -1.66 |

**Supplemental Table 8.** State based AAMR for chronic lower respiratory diseases related mortality.

| **State** | **Rank** | **Percentile** | **AAMR per 100,000** |
| --- | --- | --- | --- |
| Hawaii | 1 | 0 | 29.5 |
| District of Columbia | 2 | 2 | 37.2 |
| New Jersey | 3 | 4 | 47.4 |
| New York | 4 | 6 | 47.9 |
| Connecticut | 5 | 8 | 50 |
| Utah | 6 | 10 | 51.3 |
| Maryland | 7 | 12 | 52.2 |
| Massachusetts | 8 | 14 | 52.5 |
| Minnesota | 9 | 16 | 55 |
| California | 10 | 18 | 56.5 |
| Rhode Island | 11 | 20 | 56.8 |
| North Dakota | 12 | 22 | 57.7 |
| Pennsylvania | 13 | 24 | 58.6 |
| Virginia | 14 | 26 | 59 |
| Illinois | 15 | 28 | 59.5 |
| Florida | 16 | 30 | 59.8 |
| Wisconsin | 17 | 32 | 59.9 |
| Alaska | 18 | 34 | 62.1 |
| Delaware | 19 | 36 | 63.5 |
| Washington | 20 | 38 | 63.5 |
| Texas | 21 | 40 | 64.6 |
| Louisiana | 22 | 42 | 65.5 |
| Oregon | 22 | 42 | 66.8 |
| New Hampshire | 24 | 46 | 66.9 |
| South Dakota | 25 | 48 | 67.1 |
| Arizona | 26 | 50 | 67.3 |
| Michigan | 27 | 52 | 68.6 |
| Vermont | 28 | 54 | 68.6 |
| North Carolina | 29 | 56 | 69.9 |
| New Mexico | 30 | 58 | 70.2 |
| Iowa | 31 | 60 | 70.5 |
| Georgia | 32 | 62 | 70.8 |
| South Carolina | 33 | 64 | 71.7 |
| Idaho | 34 | 66 | 72.4 |
| Colorado | 35 | 68 | 74 |
| Nebraska | 36 | 70 | 74 |
| Maine | 37 | 72 | 75.1 |
| Ohio | 38 | 74 | 76 |
| Kansas | 39 | 76 | 76.2 |
| Missouri | 40 | 78 | 76.9 |
| Montana | 41 | 80 | 81.1 |
| Tennessee | 42 | 82 | 81.3 |
| Alabama | 43 | 84 | 82 |
| Mississippi | 44 | 86 | 82.5 |
| Nevada | 45 | 88 | 82.8 |
| Indiana | 46 | 90 | 83.7 |
| Arkansas | 47 | 92 | 85.5 |
| Wyoming | 48 | 94 | 89.7 |
| Kentucky | 49 | 96 | 94.1 |
| Oklahoma | 50 | 98 | 94.2 |
| West Virginia | 51 | 100 | 95.1 |

**Supplemental Table 9.** State based change in AAMR from 1999 to 2020 for chronic lower respiratory diseases related mortality.

| State | 1999 | 2009 | 2019 | 2020 | Change in AAMR per 100,000 from 1999 to 2020 |
| --- | --- | --- | --- | --- | --- |
| Alaska | 87.2 | 76.6 | 46.9 | 47 | -40.2 |
| Wyoming | 116.9 | 88.2 | 79.9 | 82.4 | -34.5 |
| Washington | 79 | 68.5 | 52.4 | 45.7 | -33.3 |
| Nevada | 99.9 | 82.2 | 72.8 | 69.1 | -30.8 |
| California | 72.3 | 57.8 | 44.7 | 43.4 | -28.9 |
| Colorado | 89.5 | 73.5 | 62.9 | 60.6 | -28.9 |
| New Hampshire | 78.5 | 70.5 | 60.4 | 51.7 | -26.8 |
| Montana | 91.7 | 79.7 | 78.5 | 65.4 | -26.3 |
| Oregon | 77.9 | 70 | 58.6 | 53.4 | -24.5 |
| Arizona | 79 | 65.9 | 58.7 | 56.4 | -22.6 |
| Maryland | 64 | 55.8 | 45 | 42.7 | -21.3 |
| Massachusetts | 64.1 | 52.2 | 48.2 | 42.9 | -21.2 |
| Connecticut | 58.2 | 51.7 | 43 | 37.6 | -20.6 |
| Vermont | 75.6 | 76.3 | 57 | 57.8 | -17.8 |
| New York | 56.8 | 48.3 | 42.3 | 39.1 | -17.7 |
| New Mexico | 81.6 | 72.7 | 61.9 | 63.9 | -17.7 |
| Maine | 81.2 | 75.1 | 74.5 | 64.1 | -17.1 |
| Rhode Island | 62.7 | 61.4 | 52.6 | 45.6 | -17.1 |
| Virginia | 67.5 | 61.1 | 55.2 | 50.5 | -17 |
| New Jersey | 55.2 | 49 | 40.4 | 38.5 | -16.7 |
| Minnesota | 63.7 | 53.7 | 49.2 | 47.1 | -16.6 |
| Texas | 72.1 | 65.7 | 59.6 | 55.8 | -16.3 |
| Delaware | 66.1 | 67.1 | 54.1 | 50 | -16.1 |
| Idaho | 76.6 | 74.4 | 73.3 | 60.7 | -15.9 |
| District of Columbia | 45.9 | 37.9 | 29.3 | 31 | -14.9 |
| Florida | 66.4 | 62 | 54 | 51.6 | -14.8 |
| North Carolina | 74 | 69.9 | 64 | 59.4 | -14.6 |
| Illinois | 66.4 | 62 | 53.8 | 52.3 | -14.1 |
| Pennsylvania | 63.4 | 61.1 | 52.7 | 50.6 | -12.8 |
| Georgia | 75.6 | 72 | 64.8 | 64 | -11.6 |
| Iowa | 72.2 | 75.1 | 67.5 | 60.7 | -11.5 |
| Nebraska | 77.7 | 74.6 | 71.1 | 66.8 | -10.9 |
| Hawaii | 36.9 | 29.6 | 27.3 | 27.4 | -9.5 |
| Ohio | 76.9 | 77.7 | 70.7 | 68.8 | -8.1 |
| Utah | 58.2 | 45.6 | 48 | 50.6 | -7.6 |
| Wisconsin | 62.3 | 59.5 | 56.7 | 54.7 | -7.6 |
| Kansas | 75 | 78 | 74.1 | 67.8 | -7.2 |
| Missouri | 79.6 | 79.9 | 72 | 72.4 | -7.2 |
| Michigan | 69.7 | 69.3 | 66.8 | 64.4 | -5.3 |
| South Carolina | 72.4 | 74.1 | 66.5 | 67.2 | -5.2 |
| Kentucky | 91.1 | 96.1 | 96.2 | 86.6 | -4.5 |
| South Dakota | 59.5 | 69.4 | 69.7 | 56.7 | -2.8 |
| North Dakota | 55.1 | 64 | 53.9 | 53.4 | -1.7 |
| Tennessee | 77 | 81.6 | 82 | 78.7 | 1.7 |
| Louisiana | 61.9 | 65.7 | 59.6 | 63.9 | 2 |
| West Virginia | 89.4 | 98.6 | 95.8 | 91.6 | 2.2 |
| Indiana | 79.3 | 85.4 | 86.3 | 82.9 | 3.6 |
| Alabama | 75 | 83.6 | 85.6 | 81.7 | 6.7 |
| Oklahoma | 75.9 | 100.3 | 96.3 | 92.5 | 16.6 |
| Mississippi | 72.8 | 81.1 | 87.5 | 91.2 | 18.4 |
| Arkansas | 72 | 86.4 | 92.6 | 93.2 | 21.2 |

**Supplemental Table 10.** Focused state analysis of chronic lower respiratory diseases related mortality in the United States.

| Year | Alaska | Wyoming | Washington | Arkansas | West Virginia | District of Columbia | Hawaii |
| --- | --- | --- | --- | --- | --- | --- | --- |
| 1999 | 87.2 | 116.9 | 79 | 72 | 89.4 | 45.9 | 36.9 |
| 2000 | 73.4 | 96.3 | 76.2 | 73.7 | 96.9 | 48.4 | 34.5 |
| 2001 | 77.1 | 88.7 | 74.2 | 71.2 | 91.6 | 41.2 | 33.7 |
| 2002 | 73.5 | 106.2 | 75.1 | 74.6 | 87.2 | 36.3 | 31 |
| 2003 | 72.2 | 88.8 | 72 | 77.3 | 91.2 | 38.1 | 32.4 |
| 2004 | 60.9 | 96.7 | 67.6 | 72.9 | 85.8 | 44.9 | 33.9 |
| 2005 | 66 | 89.1 | 70.1 | 78.6 | 93.6 | 36.9 | 30.6 |
| 2006 | 58.4 | 104.2 | 67.2 | 74.1 | 87 | 35.4 | 30.7 |
| 2007 | 70.3 | 87.4 | 66.1 | 80.9 | 89.8 | 35.9 | 30.7 |
| 2008 | 68.9 | 90.2 | 70.3 | 90.7 | 106.4 | 39 | 29.7 |
| 2009 | 76.6 | 88.2 | 68.5 | 86.4 | 98.6 | 37.9 | 29.6 |
| 2010 | 63.7 | 91.1 | 62.5 | 82.6 | 96.8 | 39.1 | 27.5 |
| 2011 | 64.6 | 88.1 | 67.9 | 90.8 | 102.2 | 39.2 | 29.1 |
| 2012 | 63.2 | 86.2 | 63.3 | 86 | 96.1 | 35.7 | 27.4 |
| 2013 | 58.4 | 97.1 | 60.8 | 92.7 | 99.6 | 38.7 | 24 |
| 2014 | 59.2 | 84.8 | 58.5 | 90.6 | 97.1 | 33.8 | 26.4 |
| 2015 | 58.2 | 87.7 | 61.3 | 96.2 | 99.7 | 35.7 | 26.8 |
| 2016 | 64.2 | 75 | 57.4 | 90.9 | 95.7 | 36.9 | 27.4 |
| 2017 | 55.5 | 83.2 | 58.7 | 102.6 | 99.4 | 30.4 | 29.3 |
| 2018 | 54.6 | 90.7 | 53.2 | 95.4 | 99.3 | 34.6 | 29.9 |
| 2019 | 46.9 | 79.9 | 52.4 | 92.6 | 95.8 | 29.3 | 27.3 |
| 2020 | 47 | 82.4 | 45.7 | 93.2 | 91.6 | 31 | 27.4 |
| **Number of Joinpoints (years of Joinpoint)** | 0 | 0 | 1 (2017) | 1 (2017) | 0 | 0 | 1 (2013) |
| **APC-Segment 1 (95% CI)** | -2.02 | -1.06 | -1.57 | 1.85 | 0.38 | -1.53 | -2.09 |
| **APC-Segment 2 (95% CI)** |  |  | -6.95 | -2.02 |  |  | 1.13 |
| **Average APC** | -2.02 | -1.06 | -2.36 | 1.29 | 0.38 | -1.53 | -1.03 |

**Supplemental Table 11.** Chronic lower respiratory diseases related mortality rates stratified by

urban and rural classification in the United States.

| Year | Rural Overall | Rural Female | Rural Male | Urban Overall | Urban Female | Urban Male |
| --- | --- | --- | --- | --- | --- | --- |
| 1999 | 76.3 | 57.9 | 106.7 | 68.6 | 58.2 | 86.6 |
| 2000 | 74.9 | 57.9 | 103 | 66.5 | 57.6 | 81.9 |
| 2001 | 75.9 | 60.1 | 102 | 65.7 | 57.8 | 79.5 |
| 2002 | 77.1 | 61.8 | 102.4 | 65.5 | 57.3 | 79.3 |
| 2003 | 77.2 | 63.3 | 100 | 65.2 | 57.9 | 77.6 |
| 2004 | 73.4 | 60.3 | 94.8 | 62.1 | 55.2 | 73.6 |
| 2005 | 79.4 | 65.7 | 100.9 | 65.1 | 58.4 | 75.9 |
| 2006 | 74.1 | 62.4 | 92.8 | 60.8 | 54.9 | 70.6 |
| 2007 | 76.9 | 64.6 | 96.2 | 60.8 | 54.6 | 70.5 |
| 2008 | 83.6 | 70.7 | 103.8 | 65.6 | 59.5 | 75.4 |
| 2009 | 81.1 | 69.7 | 98.6 | 62.4 | 56.8 | 71.3 |
| 2010 | 80.2 | 69.6 | 96.7 | 61.7 | 56.3 | 70.3 |
| 2011 | 82 | 71.7 | 97.5 | 62 | 56.9 | 69.9 |
| 2012 | 81.9 | 72.4 | 96.2 | 60.3 | 55.5 | 67.7 |
| 2013 | 82.8 | 73.3 | 96.9 | 61.2 | 56.7 | 68 |
| 2014 | 80.8 | 72.6 | 92.8 | 58.6 | 54.2 | 65 |
| 2015 | 84.7 | 76.3 | 96.8 | 59.9 | 56.2 | 65.3 |
| 2016 | 82.5 | 74.3 | 94.2 | 58.4 | 54.4 | 64.3 |
| 2017 | 84.8 | 77.6 | 95.3 | 58.8 | 55.2 | 63.9 |
| 2018 | 82.7 | 75.2 | 93 | 57 | 53.2 | 62 |
| 2019 | 81 | 74.5 | 89.8 | 54.5 | 51.3 | 58.8 |
| 2020 | 78.4 | 71.7 | 87.4 | 51.7 | 48 | 56.7 |
| **Number of Joinpoints (years of Joinpoint)** | 1 (2017) | 1 (2017) | 0 | 1 (2017) | 1 (2017) | 1 (2018) |
| **APC-Segment 1 (95% CI)** | 0.67 | 1.64 | -0.6 | -0.74 | -0.23 | -1.49 |
| **APC-Segment 2 (95% CI)** | -2.28 | -2.57 |  | -3.79 | -4.26 | -4.59 |
| **Average APC** | 0.25 | 1.03 | -0.6 | -1.18 | -0.81 | -1.79 |

**Supplemental Figure 1.** APC for overall and gender stratified chronic lower respiratory diseases related mortality rate in the United States from 1999 to 2020. *Indicates the APC is significantly different from 0.


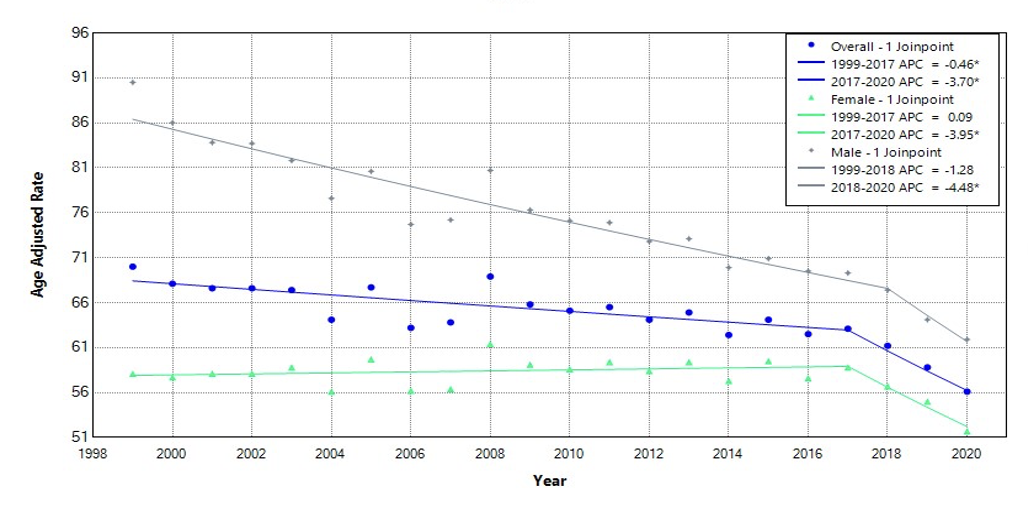


**Supplemental Figure 2a.** Data regarding mortality rates associated with chronic lower respiratory diseases in the United States between 1999 and 2020 for different racial/ethnic groups. *Indicates the APC is significantly different from 0.

**Supplemental Figure 2b.** Join point models for stratified chronic lower respiratory diseases related mortality rate in the United States from 1999 to 2020 in various ethnic/racial groups. *Indicates the APC is significantly different from 0.
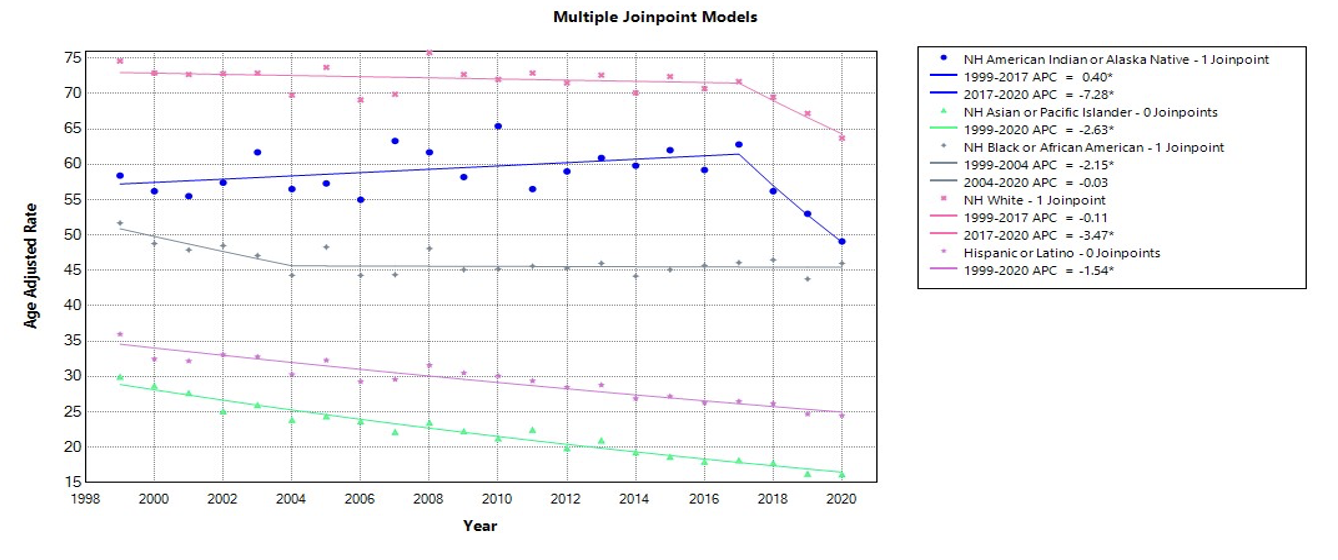


**Supplemental Figure 3a.** Joinpoint models stratified by racial/ethnic groups and male gender for chronic lower respiratory diseases related mortality rate in the United States from 1999 to 2020. *Indicates the APC is significantly different from 0.


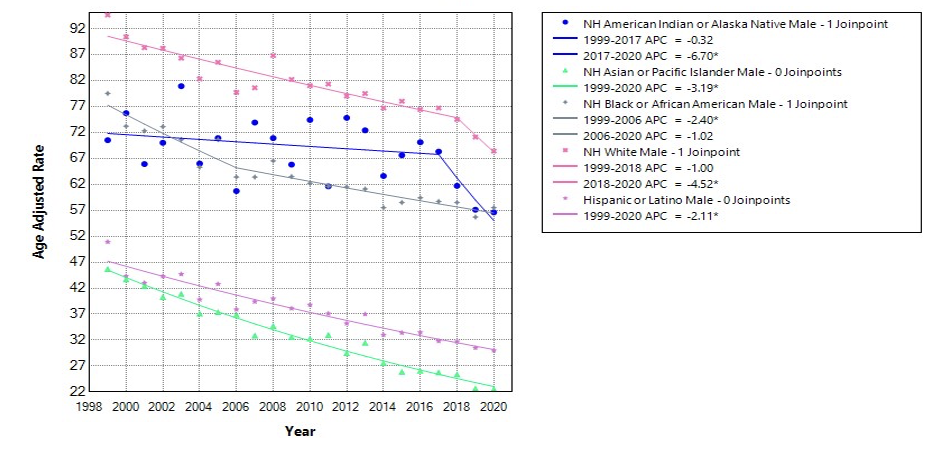


**Supplemental Figure 3b.** Joinpoint models stratified by racial/ethnic groups and female gender for chronic lower respiratory diseases related mortality rate in the United States from 1999 to 2020. *Indicates the APC is significantly different from 0.


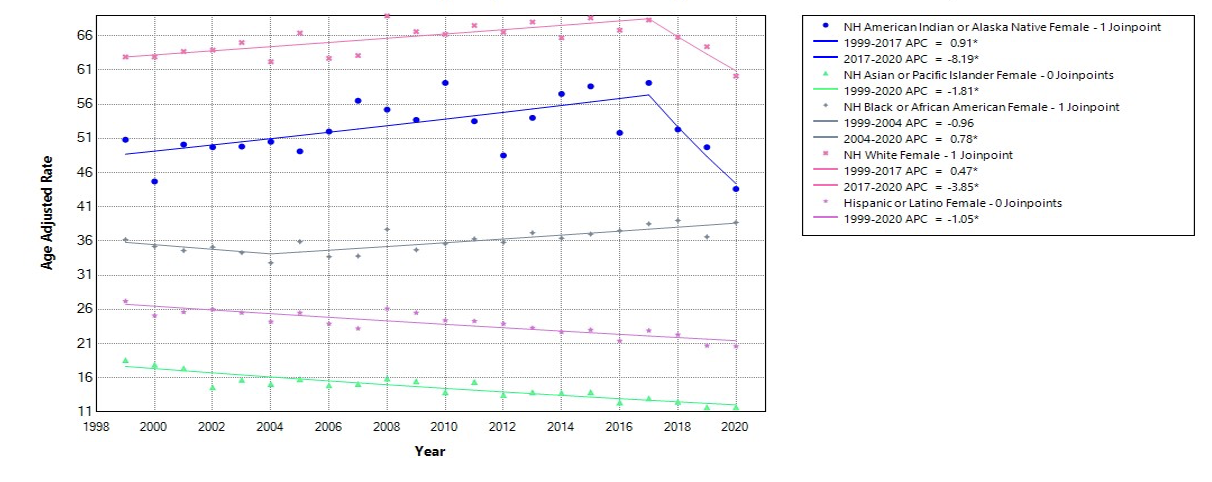


**Supplemental Figure 4a.** Data stratified for crude mortality rate in different age groups for chronic lower respiratory diseases related mortality in the United States between 1999 and 2020. *Indicates the APC is significantly different from 0.

**Supplemental Figure 4b.** Joinpoint models stratified by age groups for chronic lower respiratory diseases related mortality rate in the United States from 1999 to 2020. *Indicates the APC is significantly different from 0.


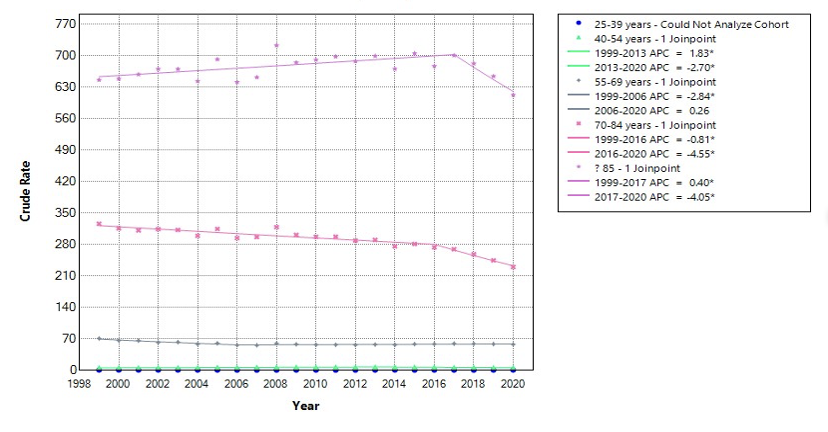


**Supplemental Figure 5a.** Data stratified by region for chronic lower respiratory diseases related mortality in the United States between 1999 and 2020. *Indicates the APC is significantly different from 0.

**Supplemental Figure 5b.** Joinpoint models stratified by region for chronic lower respiratory diseases related mortality rate in the United States from 1999 to 2020. *Indicates the APC is significantly different from 0.


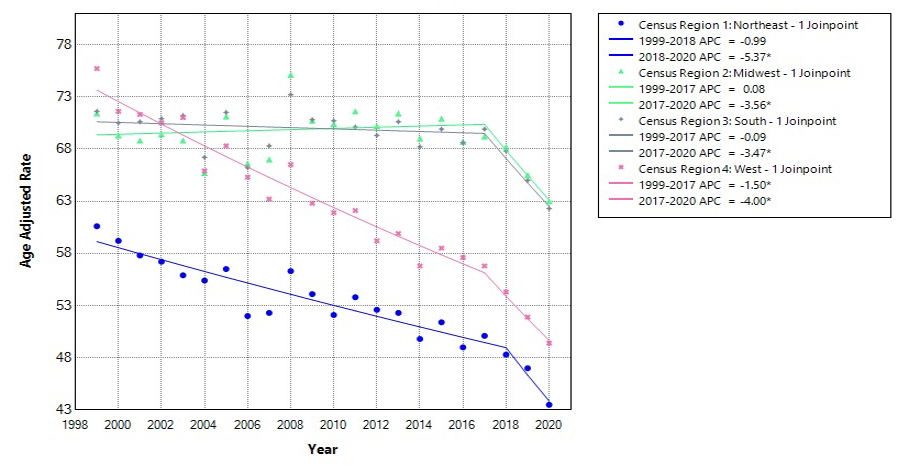


**Supplemental Figure 6a.** Joinpoint models stratified by region and female gender for chronic lower respiratory diseases related mortality rate in the United States from 1999 to 2020. *Indicates the APC is significantly different from 0.


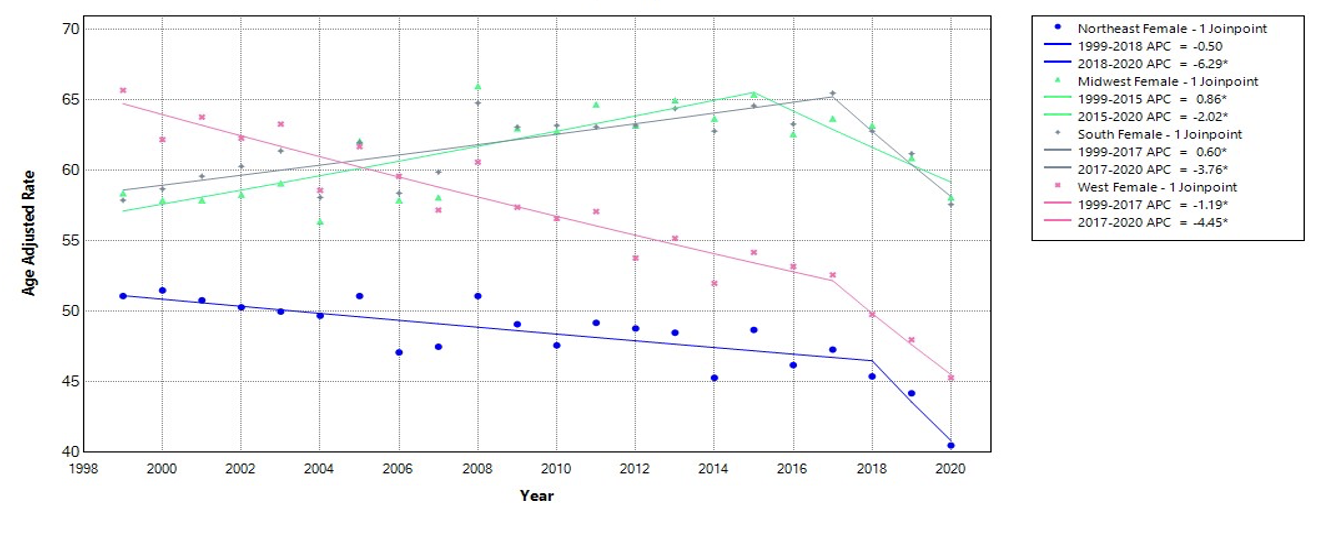


**Supplemental Figure 6b.** Joinpoint models stratified by region and male gender for chronic lower respiratory diseases related mortality rate in the United States from 1999 to 2020. *Indicates the APC is significantly different from 0.


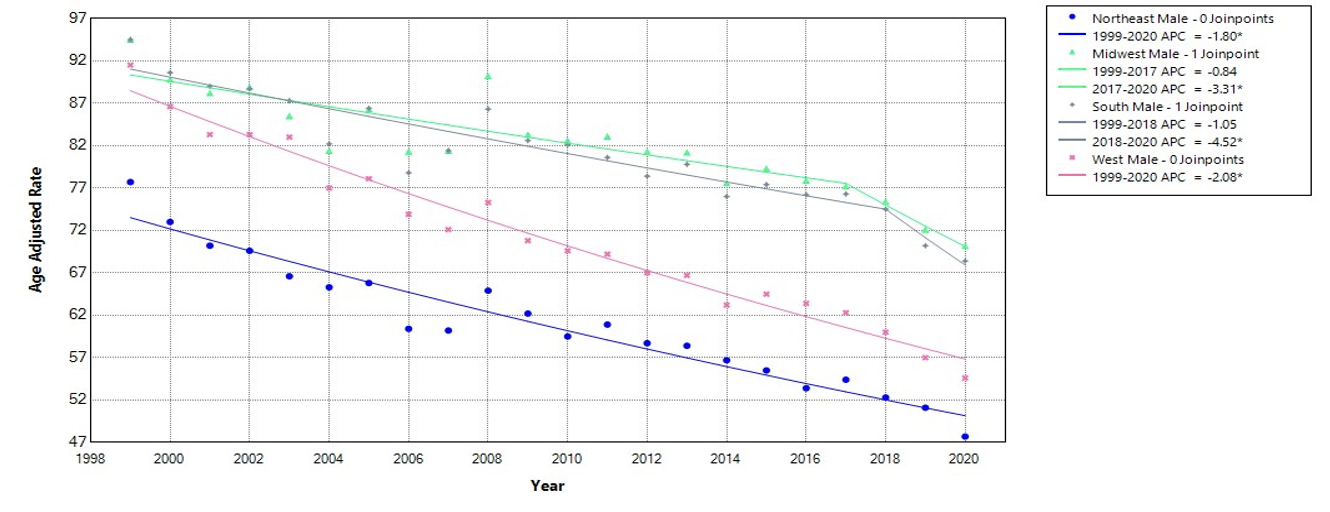


**Supplemental Figure 7a.** Data stratified by state regarding mortality rates associated with chronic lower respiratory diseases in the United States.


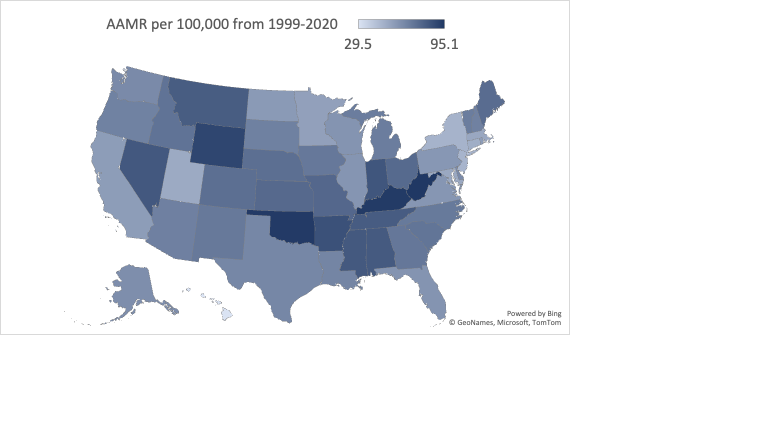


**Supplemental Figure 7b.** State based changes in AAMR per 100,000 between the years of 1999 and 2020 for chronic lower respiratory diseases related mortality in the United States

**Supplemental Figure 8a.** Data stratified by specific states regarding mortality rates associated with chronic lower respiratory diseases in the United States. *Indicates the APC is significantly different from 0.

**Supplemental Figure 8b.** Joinpoint models stratified by specific states for chronic lower respiratory diseases related mortality rate in the United States from 1999 to 2020. *Indicates the APC is significantly different from 0.


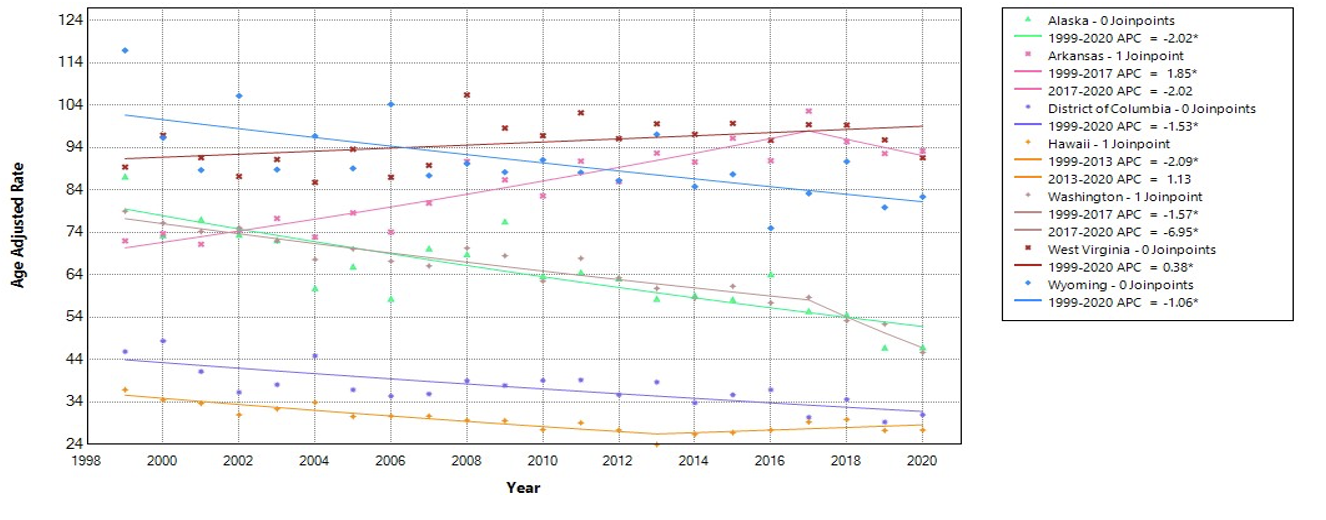


**Supplemental Figure 9.** Joinpoint models stratified by gender and rural-urban classifications for chronic lower respiratory diseases related mortality rate in the United States from 1999 to 2020. *Indicates the APC is significantly different from 0.


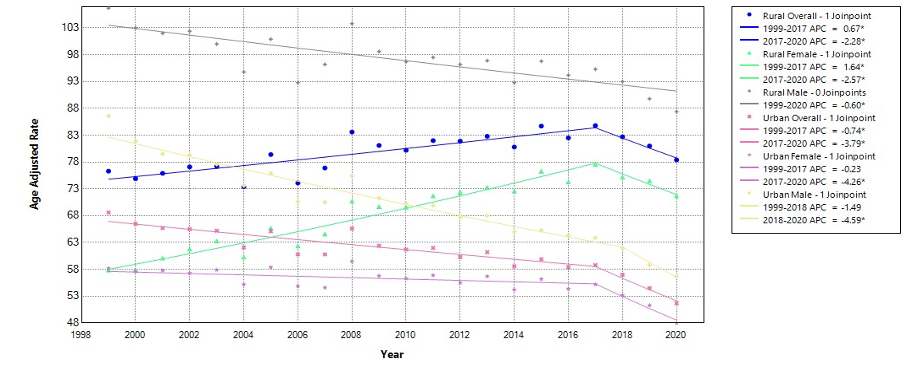

Supplement: Supplementary file 1 — Supplementary Material 1 [file 12931_2024_2880_MOESM1_ESM.docx]
